# Peer Verbal Encouragement Is More Effective than Coach Encouragement in Enhancing CrossFit-Specific 1-RM Strength, Functional Endurance, and Psychophysiological Assessment Performance

**DOI:** 10.3390/sports12030064

**Published:** 2024-02-20

**Authors:** Amir Romdhani, Faten Sahli, Omar Trabelsi, Mahmoud Rebhi, Hatem Ghouili, Hajer Sahli, Atef Salem, Khaled Trabelsi, Haitham Jahrami, Achraf Ammar, Makram Zghibi

**Affiliations:** 1 High Institute of Sport and Physical Education of Ksar Said, University of Tunis, Tunis 2009, Tunisia; romdhaniamir5@gmail.com (A.R.); sehli.feten@gmail.com (F.S.); 2 Research Unit, Sportive Performance and Physical Rehabilitation, High Institute of Sports and Physical Education of Kef, University of Jendouba, Kef 7100, Tunisia; omar.trabelsi@issepk.rnu.tn (O.T.); rebhimahmoud@gmail.com (M.R.); hatemghouili@gmail.com (H.G.); sahlihajer2005@yahoo.fr (H.S.); makwiss@yahoo.fr (M.Z.); 3 Research Unit: Physical Activity, Sport and Health, UR18JS01, National Observatory of Sport, Tunis 1003, Tunisia; 4 High Institute of Sport and Physical Education of Sfax, University of Sfax, Sfax 3000, Tunisiatrabelsikhaled@gmail.com (K.T.); 5 Department of Training and Movement Science, Institute of Sport Science, Johannes Gutenberg-University Mainz, 55122 Mainz, Germany; 6 Research Laboratory: Education, Motricity, Sport and Health, EM2S, LR19JS01, University of Sfax, Sfax 3000, Tunisia; 7 Ministry of Health, Manama 410, Bahrain; hjahrami@health.gov.bh; 8 Department of Psychiatry, College of Medicine and Medical Sciences, Arabian Gulf University, Manama 323, Bahrain; 9 Research Laboratory, Molecular Bases of Human Pathology, LR19ES13, Faculty of Medicine of Sfax, University of Sfax, Sfax 3000, Tunisia

**Keywords:** physical fitness, maximal testing, endurance training, psychophysiological assessments, motivation

## Abstract

This study compares the effects of coach verbal encouragement (CVE) and peer verbal encouragement (PVE) on CrossFit-specific one-repetition maximum (1-RM) strength, functional endurance, and psychophysiological assessments. A total of 36 sports science students (18 males, 18 females; mean age: 21.3 ± 0.5 years) participated in a randomized, counterbalanced crossover study in which 1-RM strength and endurance assessment sessions were undertaken under PVE, CVE, and no verbal encouragement (NVE) on separate days. Here, 1-RM strength was assessed through squat, deadlift, and bench press exercises, while endurance was evaluated using 8 min time trials (8MTT). Following the physical assessments, psychophysiological evaluations were conducted using the Borg Rating of Perceived Exertion (RPE) and the Feeling Scale (FS). The findings revealed that, after PVE, all the 1-RM strength test, 8MTT, RPE, and FS values exhibited significant increases compared to those of CVE (*p* [<0.001–<0.01], r = −0.84 [large]) and NVE (*p* [<0.001–<0.05], r [−0.43–0.52] [small]). Exceptions were noted in 1-RM-deadlift (*p* > 0.05, r = −0.43 [small]) and 1-RM-bench-press (*p* > 0.05, r = −0.43 [small]), where CVE demonstrated higher scores (1-RM-squat, 8MTT, RPE, and FS) (*p* [<0.001–<0.05], r = −0.64 [large]) in comparison to NVE. In conclusion, the study established that PVE is more impactful than CVE in enhancing CrossFit-specific 1-RM strength, functional endurance, and psychophysiological assessment performance. These findings suggest that coaches/teachers should consider involving their athletes in the reinforcement process for evaluated peers. This collaborative approach may not only optimize performance outcomes but also foster a supportive and motivational training environment.

## 1. Introduction

CrossFit training emphasizes maximum strength in exercises such as squat, deadlift, and bench press, as well as functional endurance qualities. Studies have shown that CrossFit competitors exhibit significant correlations between their performance in specific CrossFit workouts and measures of maximum strength and endurance [1]. In addition, a study comparing CrossFit training to traditional resistance training revealed that both modalities were equally effective at improving maximal strength, explosive strength, and endurance strength in trained adults [2]. Furthermore, a study investigating the association of anthropometrics with strength and endurance performance in resistance-trained males and females showed that lean mass and body height were associated with maximal strength, while body height was inversely associated with endurance performance [3].

Physical fitness, cognitive and technical abilities, and environmental influences are interconnected factors that play crucial roles in enhancing physical and athletic performance, as well as improving learning outcomes in sports. For instance, higher levels of physical fitness can positively impact technical execution, while increased motivation is essential for improving physical conditioning. Pacholek and Zemková [4] found that technical abilities are also an important factor in athletic performance. Verbal encouragement (VE) during maximal exercise testing has been shown to have a significant impact on performance and motivation [5].

VE plays a pivotal role in human psychology, influencing individuals’ cognitive and emotional processes. Rooted in behavioral psychology, the power of positive words and motivation is exemplified through theories. Adler [6] pioneered the concept of encouragement, deeming it crucial for human development and psychotherapy. Recognizing our innate social orientation, Adler highlighted encouragement’s role in fostering engagement, particularly when individuals lose their social interest. From another perspective, Albert Bandura’s [7] influential theory on self-efficacy outlines four sources, one of which is verbal persuasion. This latter, also termed social persuasion, includes what individuals communicate to others regarding their beliefs about capabilities, influencing and shaping these beliefs positively or negatively [7]. While Bandura’s concept of verbal persuasion incorporates both positive and negative elements, often including expressions of doubt in addition to encouragement, research studies predominantly operationalize it to focus on positive persuasion.

Studies have reported significant increases in the time to exhaustion and distance covered during various maximal exercise tests when participants received verbal encouragement [5]. Research has demonstrated that receiving VE from coaches and peers can have a positive effect on measuring maximal force [4]. In a study that compared various forms of encouragement during maximal voluntary isometric contraction, it was found that combining verbal encouragement with visual feedback led to the highest peak muscle force in both strength-trained and untrained individuals [8]. However, it is worth noting that previous studies have not specifically addressed the potential influence of sex on the impact of verbal encouragement on maximal force.

The VE from coaches and peers has been shown to have a positive impact on maximal aerobic endurance performance in athletes. Studies have reported significant increases in the time to exhaustion during VO_2_max and multistage shuttle run tests, as well as improvements in the 6 min walk test distance, when participants received verbal encouragement [5]. Additionally, external VE during both sprint and endurance activities has resulted in large improvements in performance and motivation to continue exercising [9]. VE has also been found to have a significant effect on maximal oxygen uptake, run time, heart rate, and blood lactate level during maximal running tests [10]. Overall, VE from coaches and peers can enhance maximal aerobic endurance assessment in athletes, leading to improved performance and motivation [11].

On the other hand, the effects of VE on the perceived exertion (RPE) and feeling scale (FS) measures during maximal physical tests have not been explicitly mentioned in previous studies. One study investigated the effects of the frequency of VE during maximal exercise testing but did not specifically measure RPE or FS [12]. Another study examined the effects of sex role orientation on physical exertion, revealing that feminine-typed women gave significantly higher exertional ratings than masculine or androgynous women [13]. Collectively, although VE has demonstrated benefits for enhancing performance and motivation, its impact on RPE and FS during maximal physical activity remains unclear.

The purpose of this study was to investigate the impact of coaches’ (CVE) vs. peers’ VE (PVE) on CrossFit-related physical performances and psychophysiological responses among sports science students, using measures including one-repetition maximum (1-RM), functional aerobic performance (i.e., 8 min time trials—8MTT), RPE, and FS.

## 2. Materials and Methods

### 2.1. Participants

G*power software (version 3.1.9.6; Kiel University, Kiel, Germany) was used to calculate the minimum required sample size. A sample size of 22 participants would be sufficient to detect significant differences (effect size f = 0.55, α = 0.05) with an actual power of 80%. Thirty-six sports science students (18 males and 18 females, age: 21.3 ± 0.5 years, body mass: 70.2 ± 9.3 kg, body height: 1.75 ± 0.1 m, body mass index: 22.7 ± 2.6 kg·m^−2^, body fat percentage: 23.1 ± 2.16%) from the Higher Institute of Sports and Physical Education of Kef (Kef, Tunisia) voluntarily participated in this study. The participants in this study were carefully selected based on specific inclusion criteria. Initially, individuals were chosen for their high level of physical activity; they engaged in regular physical activity for approximately 7 h per week, including 2 h of CrossFit. Additionally, participants had prior experience in relevant activities, ranging from 1 to 3 years. Before the study began, all participants completed medical questionnaires and signed written informed consent forms. Notably, none of the participants reported having any neuromuscular disorders or specific musculoskeletal injuries affecting their ankles, knees, or hips within the past year. This exclusion criterion was applied to ensure that participants did not have any conditions that could potentially bias the results or hinder their ability to accurately perform the required physical tasks.

Prior to their inclusion in the study, all participants received comprehensive verbal and written instructions that explained the procedures and potential risks involved. The participants were also informed of their right to withdraw from the trial at any point. Additionally, the requisite legal permission for access to the study site (ISSEPK) was diligently sought and obtained from all relevant stakeholders. The research project underwent a comprehensive review, received approval for implementation from the local Personal Protection Committee (PPC: No. 24/2022), and was conducted in accordance with the recommendations of the World Health Organization outlined in the Helsinki Declaration.

### 2.2. Design

The current study used a counterbalanced crossover within subject design with repeated measures design. This study involved three experimental conditions to examine the effects of providing external verbal encouragement (VE) (Coach VE, CVE; Peer VE, PVE; and No VE, NVE) on maximal physical fitness test performance (i.e., 1-RM of squat, deadlift, bench-press tests, 8MTT) and psychophysiological indices (i.e., FS, RPE) scores. Participants were randomly assigned to three groups, each experiencing a diverse sequence of VE conditions. In the initial week, Group 1 was initiated with CVC, Group 2 with PVE, and Group 3 with NVE. The following weeks, the assignments were reversed, maintaining this alternating pattern throughout the study duration. Overall, participants were asked to complete twelve experimental trials (4 physical fitness tests × 3 VE conditions) and six questionnaires (FS and RPE × 3 VE conditions) in a randomized and counterbalanced order.

### 2.3. Procedures

The investigation took place at the gym of the High Institute of Sports and Physical Education of Kef (Tunisia) during the month of March, spanning over three weeks. Experimental sessions were scheduled on Mondays, Tuesdays, and Wednesdays from 9:00 to 11:00 a.m., with one group tested per day. The counterbalancing procedure implemented in this study ensured that each of the three groups was exposed to every VE condition in a different sequence, underwent testing on all three distinct days, and performed the 1-RM strength tests in all possible orders (additional information is provided in Table 1). The ambient temperature inside the gym varied between 10 and 16 °C, depending on the time of day. Before arrival at the testing field, all participants were requested to consume their normal afternoon snacks or hydrate for testing (i.e., 1 L of water the night before and 0.5 L of water 2 h before testing) and abstain from maximal exercise and alcohol-caffeine ingestion for 72 and 48 h before testing, respectively.

During each testing session, participants were randomly assigned to perform the three 1-RM tests (i.e., bench press, squat, and deadlift) in a random order. Following this, they completed the 8MTT test and finally answered the RPE and FS questionnaires in a random order. To ensure fairness and eliminate bias, an online random number generator (research randomizer 4.0) was utilized for this purpose. A standardized 10 min warm-up was conducted before each session, consisting of dynamic stretching, cardiovascular exercises, and mobility drills to prepare the participants physically and mentally. The first 1-RM test was collected 5 min after completing the dynamic stretching routines for all testing sessions, and this was also useful for determining natural day-to-day fluctuations in performance.

### 2.4. Verbal Encouragement (VE) Conditions

Participants received VE in the Tunisian dialect from either the coach (i.e., CVE condition) or the peer (PVE). The expressions employed were standardized and included phrases such as “Good job!”, “Keep going”, “You can do it”, and “Don’t give up” (interpretive translation). Additionally, the coach/peer personalized the encouragement by addressing each student by name (e.g., “You can do it, Ahmed” or “Don’t give up, Ahmed”) once per test task. The coach/peer adopted a loud, energetic, and enthusiastic tone, ensuring that the VE was audibly clear to all the participating students.

As participants comprised sports science students, the coach, a 30-year-old male teacher, was assigned to deliver fitness classes at the institute where the study took place. Consequently, the students were already acquainted with the coach. Under PVE, the students consistently selected the peer from whom they preferred to receive encouragement.

#### Familiarization Session

A familiarization session was conducted one week prior to the main experiment. This session provided participants with sufficient time to become accustomed to the instruments and equipment to be used in the study. Anthropometric measurements, including height and weight, were recorded at the start of the orientation meeting.

During this session, an estimation procedure was employed to ascertain the starting 1-RM squat, deadlift, and bench press weights for each participant. This process involved participants performing several submaximal lifts with progressively increasing weights. Based on their performance and feedback, an appropriate starting weight was estimated, allowing participants to safely and effectively progress toward their actual 1-RM during the experimental sessions. This approach was carefully designed to ensure that participants were appropriately challenged while minimizing the risk of injury or excessive fatigue. Furthermore, having an initial estimate of participants’ strength capabilities helped avoid unnecessary attempts at nonchallenging weights, thereby optimizing the efficiency of the experimental sessions. Participants were provided with detailed explanations and demonstrations of the correct execution of each lift.

### 2.5. Data Collection

The data collection was conducted by a team of five investigators. Three members were responsible for gathering the data during the physical tests, while the remaining two focused on gathering self-reported data from participants immediately after the completion of these tests.

#### 2.5.1. One-Repetition Maximum (1-RM)

In each strength testing session, participants engaged in two sets of the 1-RM bench press, squat, and deadlift tests. The sequence of the tests followed the recommendations of Simão et al. [12], emphasizing the prioritization of exercises that activate larger muscle groups before those targeting smaller muscle groups (e.g., bench press preceding pec fly or squat preceding leg extension). This sequencing rationale is rooted in the concept that inducing fatigue in smaller muscle groups (e.g., triceps, anterior deltoid) first through single-joint exercises (e.g., triceps extension, shoulder flexion) may impede the larger muscle groups (e.g., pectoralis major) from attaining effective overload during subsequent multi-joint exercises (e.g., bench press).

Standardized rest intervals of 2 min between repetitions, 3 min between lifts, and 5 min between sets were employed during the strength testing sessions, in accordance with previous guidelines [14,15]. The conclusion of each session involved recording the maximum weight lifted by each participant for each test.

#### 2.5.2. 8 min Time Trials (8MTT)

Participants were instructed to complete as many rounds as possible of a designated set of exercises within an 8 min timeframe. The exercises included 6 repetitions of burpees, 6 repetitions of box jumps, 6 repetitions of hand release push-ups, and 10 repetitions of sit-ups. All movements performed during the endurance test adhered to the standardized range of motion outlined by the High Intensity Functional Training (HIFT) guidelines established by Feito et al. [16]. The total count of movements completed within the 8 min duration served as a metric for participants’ endurance capacity, offering valuable insights into their ability to sustain effort and maintain performance over time.

#### 2.5.3. Maximum Heart Rate (HRmax)

Heart rate was monitored at 5 s intervals throughout the 8MTT test sessions utilizing the Polar Team Sport System 2 (Polar Electro, Kempele, Finland). HR data during the test are expressed as a percentage of the HRmax.

#### 2.5.4. Rating of Perceived Exertion (RPE)

Perceived exertion was evaluated immediately after the completion of the 8MTT using the RPE Borg [17]. In this study, a modified version of Borg’s original scale [18], known as the Categorical Ratio 10-point scale (CR10), was employed. This scale allows participants to subjectively rate and convey their perceived level of effort during the intensive 8MTT on a 10-point scale ranging from “really easy” (1) to “maximum effort” (10). To enhance the accuracy of the perceived exertion ratings, facial emoticons were associated with each anchor point on the CR10 scale. Participants received instructions to overlook isolated sensations, such as leg pain or shortness of breath, and concentrate on their overall perception of exertion during the 8MTT.

#### 2.5.5. Feeling Scale (FS)

The feeling scale (FS), as described by Hardy and Rejeski [19], was employed to assess affective valence. Participants were asked to rate their feelings following physical exercises, indicating their basic affective sensations ranging from displeasure to pleasure. Using an 11-point bipolar scale, participants provided a single-item evaluation, with values ranging from very bad (−5) to very good (+5). The scale also included neutral (0) as well as all odd integers, encompassing bad (−3), somewhat bad (−1), somewhat good (+1), and good (+3) as anchor points. The scale’s anchor points were translated from English to Arabic (the participants’ native language) by three experienced English–Arabic interpreters.

#### 2.5.6. Statistical Analysis

Data analyses were performed using SPSS version 28.0 for Windows (SPSS, Inc., Chicago, IL, USA). The values are presented as means ± standard deviations. The normality and homogeneity of the data were checked with the Kolmogorov–Smirnov and Levene’s tests, respectively. A one-way ANOVA with repeated measures was performed to analyze the effect of VE conditions on test performance. In cases where Mauchly’s assumption of sphericity was violated, the Greenhouse–Geisser correction was applied. After confirming a significant main effect, a Bonferroni-adjusted pairwise post hoc test was performed [20]. When the assumption of normality was not met, Friedman’s ANOVA was employed to compare the effects of different conditions. Upon identifying significant differences with Friedman’s ANOVA, a Wilcoxon Signed-Rank test was utilized for post hoc pairwise comparisons. To prevent type II errors in the ANOVA tests, we estimated the statistical power and effect size using the ώ and η*p*^2^ values, respectively. The Cohen scale was used to interpret “η*p*^2^”: η*p*^2^ values less than 0.06 were regarded as indicative of a small relationship, values ranging from 0.06 to less than 0.14 were categorized as moderate, and values equal to or exceeding 0.14 signified a large relationship [21]. For pairwise comparisons of the parametric data, Cohen’s *d* effect size was calculated and interpreted as follows: “trivial” (<0.2), “small” (>0.2–0.6), “moderate” (>0.6–1.2), “large” (>1.2–2), or “very large” (>2) [21]. r was used as the effect size for pairwise comparisons of nonparametric data. The r values were interpreted as small (0.1), medium (0.3), or large (0.5). A *p* value < 0.05 indicated statistical significance for all the tests.

## 3. Results

### 3.1. One-Repetition Maximum (1-RM) Assessments

#### 3.1.1. Squat

**Figure 1:**
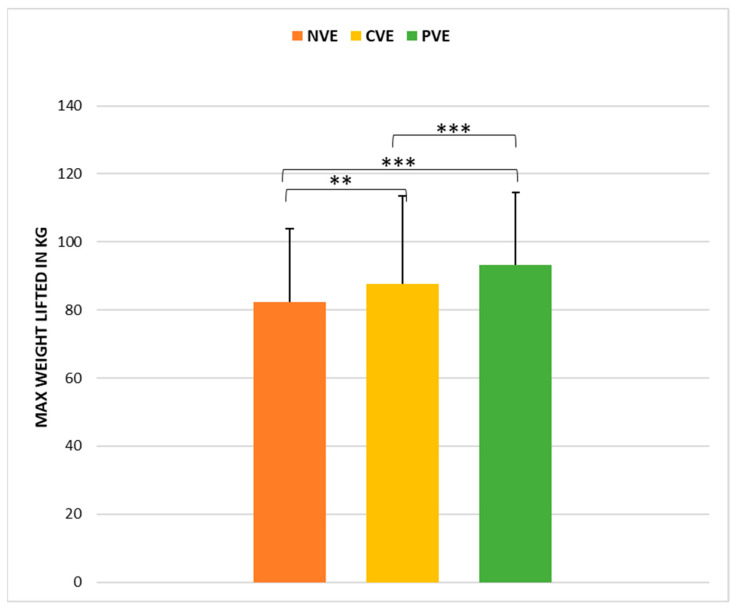
Comparison of weight lifted in the 1-RM squat test under no verbal encouragement (NVE), coach verbal encouragement (CVE), and peer verbal encouragement (PVE). **, significant difference at *p* < 0.01; ***, significant difference at *p* < 0.001.

The statistical analysis indicated the existence of a significant effect of the VE condition (*p* < 0.001, ώ = 0.49) on squat performance. Post hoc pairwise comparisons (Figure 1) revealed greater squat performance following PVE compared to CVE (z = −3.56, *p* < 0.001, r = −0.64, [large]), and following CVE compared to NVE (z = −3.14, *p* < 0.01, r = 0.52, [large]).

#### 3.1.2. Deadlift

**Figure 2:**
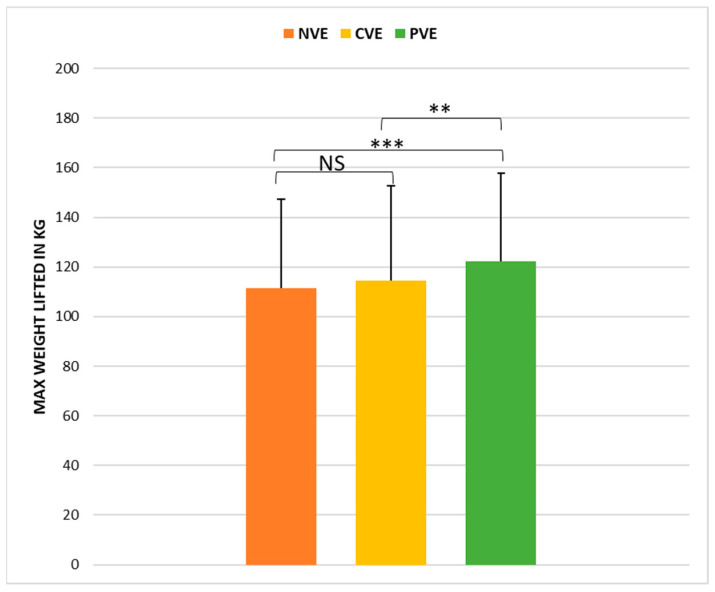
Comparison of weight lifted in the 1-RM deadlift test under no verbal encouragement (NVE), coach verbal encouragement (CVE), and peer verbal encouragement (PVE). **, significant difference at *p* < 0.01; ***, significant difference at *p* < 0.001; NS, not significant.

The statistical analysis indicated a significant effect of the VE condition (*p* < 0.001, ώ = 0.47) on deadlift performance. Further analysis through post hoc pairwise comparisons (Figure 2) demonstrated that the deadlift performances recorded following PVE were significantly greater than those following CVE (z = −3.05, *p* < 0.001, r = −0.64; [large]) and NVE (z = −4.54, *p* < 0.01; r = −0.84; [large]). There was no significant difference between the CVE and NVE conditions (z = −1.80, *p* > 0.05; r = −0.43 [medium]).

#### 3.1.3. Bench Press

**Figure 3:**
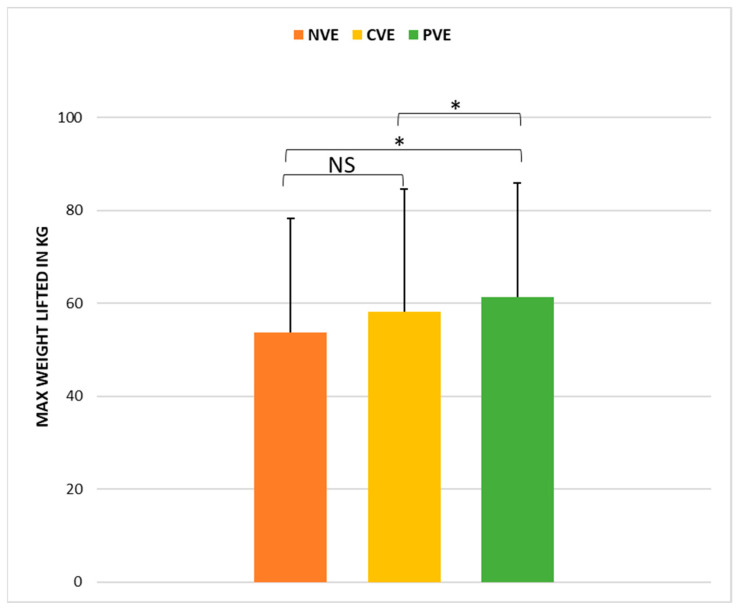
Comparison of weight lifted in the 1-RM bench press test under no verbal encouragement (NVE), coach verbal encouragement (CVE), and peer verbal encouragement (PVE). *, significant difference at *p* < 0.05; NS, not significant.

The statistical analysis indicated a significant effect of the VE condition (*p* < 0.001, ώ = 0.22) on bench press performance. Further examination via post hoc pairwise comparisons (Figure 3) demonstrated that bench press performances following PVE were significantly greater than those following CVE (z = −2.17, *p* < 0.05; r = 0.64 [large]) and NVE (z = −0.83, *p* > 0.05; r = −0.43 [small]). In contrast, there was no significant difference between the CVE and NVE conditions (z = −0.83, *p* > 0.05; r = −0.43 [small]).

### 3.2. 8 min Time Trials (8MTT) Assessment

**Figure 4:**
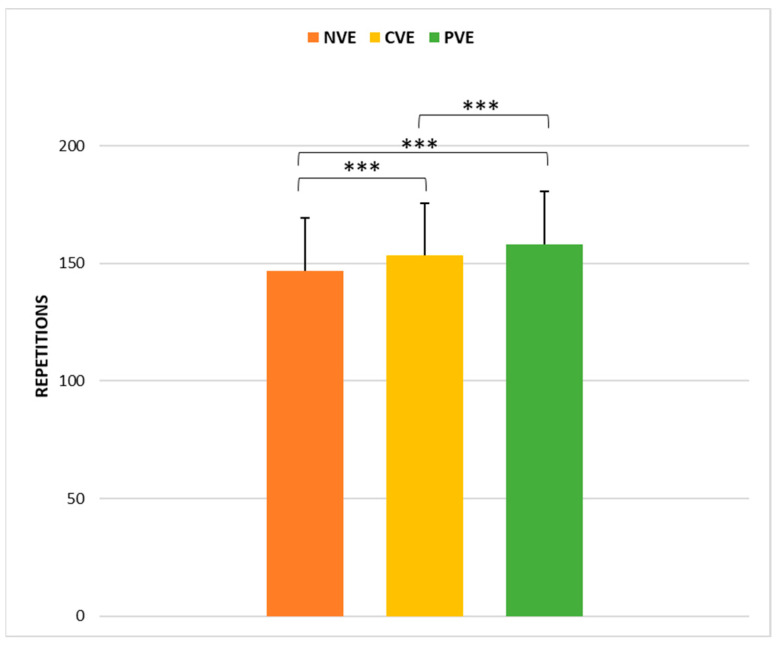
Comparison of repetitions in the 8MTT endurance test under no verbal encouragement (NVE), coach verbal encouragement (CVE), and peer verbal encouragement (PVE). ***, a significant difference at *p* < 0.001.

A statistical analysis showed a significant effect of VE conditions (*p* < 0.001, ώ = 0.65) on the 8MMT performance. Post hoc comparisons (Figure 4) revealed that performances following CVE were significantly greater than those following NVE (z = −4.80, *p* < 0.001; r = −0.43 [medium]) and significantly lower than those following PVE (z = −5.20, *p* < 0.001; r = −0.84 [large]).

### 3.3. Maximum Heart Rate (HR)

**Figure 5:**
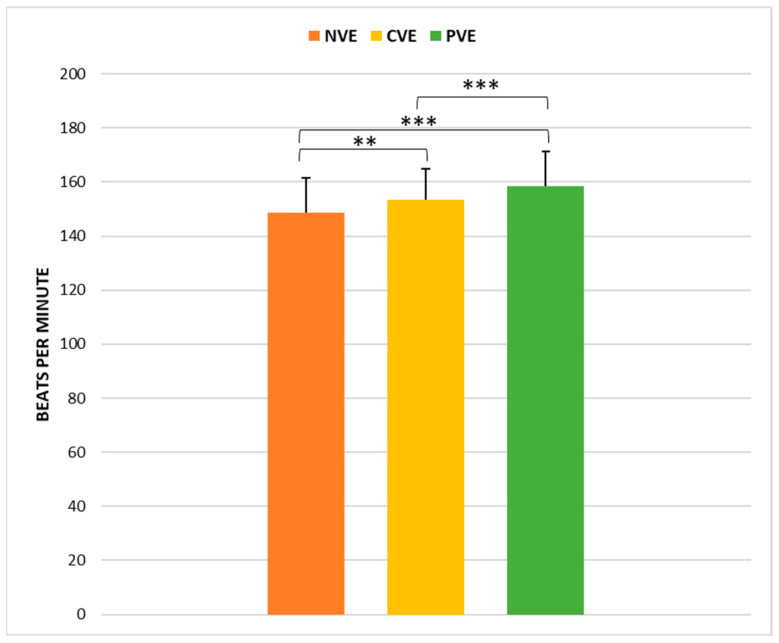
Comparison of maximum heart rate (HR) under no verbal encouragement (NVE), coach verbal encouragement (CVE), and peer verbal encouragement (PVE). **, significant difference at *p* < 0.01; ***, significant difference at *p* < 0.001.

One-way repeated-measures ANOVA revealed a significant effect of condition (F(2,70) = 33.38, *p* < 0.001; η*p*^2^ = 0.49 [large]) on maximum HR. Post hoc pairwise comparisons revealed that HR values recorded at the end of the CVE 8-MTT (153.5 ± 11.52 bpm) were significantly greater (*p* < 0.01) than those recorded at the end of the NVE (148.72 ± 12.7 bpm). Similarly, HR values recorded at the end of the PVE 8-MTT (158.47 ± 10.74 bpm) were significantly higher (*p* < 0.001) than those recorded at the end of the NVE session and those recorded at the end of the CVE session (*p* < 0.001) (Figure 5).

### 3.4. Rating of Perceived Exertion (RPE)

**Figure 6:**
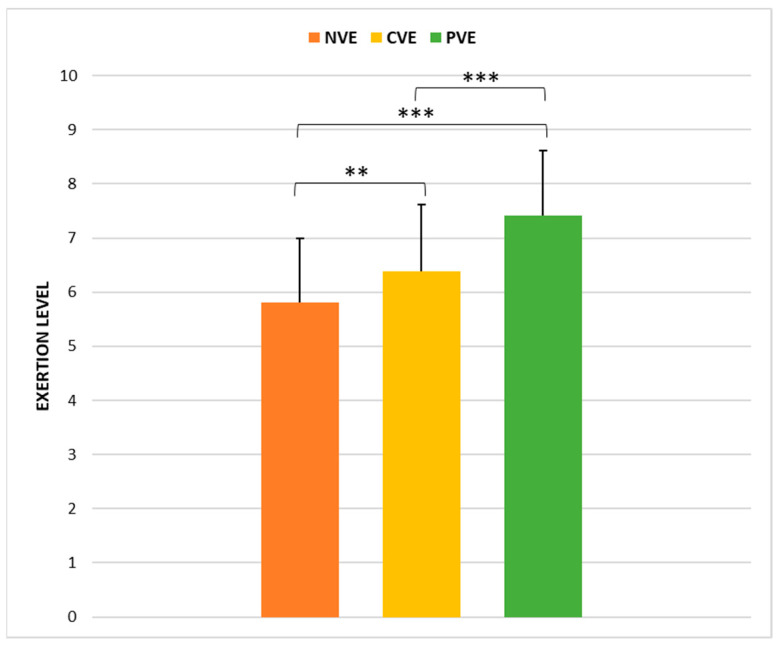
Comparison of ratings of perceived exertion (RPE) under no verbal encouragement (NVE), coach verbal encouragement (CVE), and peer verbal encouragement (PVE). **, significant difference at *p* < 0.01; ***, significant difference at *p* < 0.001.

The statistical analysis indicated a significant effect of condition (X^2^F(2) = 41.25, *p* < 0.001; ώ = 0.57) on RPE scores. Figure 6 shows the pairwise comparisons, revealing that the RPE scores collected following CVE were significantly higher than those collected following NVE (*p* < 0.01; r = −0.43 [small]), but lower compared to those collected after PVE (*p* < 0.001; r = −0.84 [large]). RPE scores collected after PVE were significantly higher compared to those after NVE (*p* < 0.001; r = −0.64 [large]).

### 3.5. Feeling Scale (FS)

**Figure 7:**
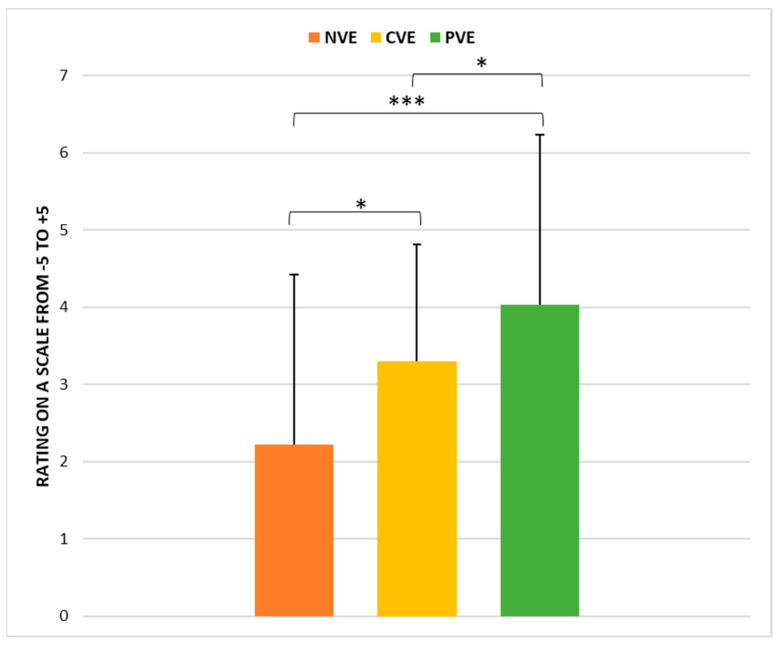
Comparison of feeling scale (FS) scores under no verbal encouragement (NVE), coach verbal encouragement (CVE), and peer verbal encouragement (PVE) conditions. *, significant difference at *p* < 0.05; ***, significant difference at *p* < 0.001.

The statistical analysis revealed a significant effect of condition (*p* < 0.001, ώ = 0.21) on FS scores. Post hoc pairwise comparisons showed that scores collected following CVE were significantly higher than those collected following NVE (*p* < 0.05; r = −0.43 [small]) and PVE (*p* < 0.001; r = −0.84 [large]) and lower compared to those collected following PVE (*p* < 0.05; r = −0.64 [large]) (Figure 7).

## 4. Discussion

The purpose of this study was to investigate the influence of verbal encouragement from coaches (CVE) and peers (PVE) on physical performance and psychophysiological indices in the context of physical fitness-related CrossFit assessment. The study showed that PVE during one-repetition-maximum (1-RM) strength tests (i.e., squat, deadlift, bench press) and functional endurance tests (e.g., 8 min time trials [8MTT]) had a positive impact on the participants’ performance. The study also revealed that CVE had a more significant impact on 1-RM-squat and 8MTT scores compared to situations where VE was lacking (NVE). Additionally, the study showed that the rates of perceived exertion (RPE) and feeling scale (FS) scores were significantly greater after PVE than after CVE. Similarly, after the CVE, these scores were significantly greater than those after the NVE.

In line with previous findings [22,23,24], the present results suggest that participants are more likely to exert their maximum effort and exhibit increased engagement in the prescribed exercises when they receive words of encouragement and motivation from their peers, as opposed to coaches. The present results also indicate that the effects of participants’ verbal inter-encouragement (i.e., PVE) are significantly greater than those of CVE in terms of 1-RM-squat, 1-RM-deadlift, 1-RM-bench-press, and 8-MTT tests (6.30%, 6.69%, 5.43%, and 3.04%, respectively). This difference can be attributed to the fact that participants feel freer and more spontaneous with VE than their peers. According to a study by Zghibi et al. [25], participants who participate in discussions in the absence of their coach display richer discourse with more developed responses. These findings suggest that encouragement from fellow students has the potential to induce a higher level of physical commitment among players, contributing to the maintenance of a sustained effort level during the proposed exercises.

Analogous studies have reported comparable outcomes. McNair et al. [26] investigated the impact of VE on the maximal force of the elbow flexor during an isometric muscle test and revealed a significant difference in favor of the VE condition. Similarly, Hammami et al. [27] explored the effects of CVE on physical performance, technical learning, and physical responses during reduced soccer games, with the findings demonstrating beneficial effects. Consequently, these authors advocate for the utilization of VE to enhance sports and motor performance. According to several studies, PVE and CVE have been shown to have positive effects on physical performance and motivation [28,29,30]. These tools are crucial for improving the teaching-learning process and motivating athletes during physical exercise [28]. The objective is to consistently motivate athletes and encourage them to push their limits in accomplishing motor tasks [31].

The RPE scores recorded after CVE were notably higher than those observed after NVE and PVE. Specifically, the scores following CVE were 6.39 ± 1.23, significantly surpassing the scores after NVE (5.81 ± 1.19) and PVE (7.42 ± 0.91). Additionally, the FS index after CVE (3.30 ± 1.51) was significantly greater than the scores after NVE (2.22 ± 2.20) and after PVE (4.03 ± 1.03). The results concerning RPE align with those found by Aydi et al. [31] and Sahli et al. [32], emphasizing the advantages of VE as a means of stimulating and motivating students during physical exercise. The VE has proven to be a valuable tool for enhancing motivation during physical exercise. This encouragement can serve as both extrinsic and intrinsic motivation, driving participants to overcome challenges faced during their exercise sessions. It has the potential to reinforce intrinsic motivation, influence physical engagement, foster positive interaction with physical effort, and cultivate a desire to exercise [31]. In alignment with this perspective, Sahli et al. [32] emphasized the importance of the CVE for improving motivation and establishing a learning climate filled with pleasure and enjoyment. Additionally, the authors showed the beneficial effects of VE and feedback on physical performance and psychophysiological responses during repeated sprint tests [32]. The findings of another study conducted by Sahli et al. [22] highlighted the substantial role of coach feedback, especially in the form of encouragement, in influencing the RPE during exercise [24]. Another study demonstrated the substantial influence of VE from both coaches and peers not only on the physical intensity exhibited by players during exercise but also on their psychophysiological responses, including mood state, physical enjoyment, and perception of effort [29]. Ruzek et al. [33] noted that interactions with peers can reinforce the representations of teammates and motivate them to push their limits during exercise. Additionally, Ruzek et al. [33] reported that emotional support from teachers and peers could give students more desire to perform physical exercise, which could explain the positive effect of VE on strength [31]. Recently, Radil et al. [34] reported that VE serves as a means of motivating participants and creating a favorable environment for the teaching-learning process.

Based on the findings of the current study, a practical recommendation for coaches and trainers is to incorporate both CVE and PVE in their athletes’ training regimens. Both PVE and CVE are effective ways to motivate students to achieve better results, especially during intense activities. Specifically, words of encouragement and motivation from teammates create a climate of peer-to-peer interaction marked by emotional support and positive reinforcement. This further fosters supportive and encouraging learning and helps students become involved and committed to the activity. Encouragement from the coach, and particularly between trainees, plays a direct role in developing a sense of confidence and a better emotional state, which translates into greater motivation and commitment to the exercise. PVE and CVE are important factors for promoting a better emotional state during the training-learning process. These findings have important implications for the design of fitness assessment programs in CrossFit and other similar contexts. Future research could explore the mechanisms underlying the effectiveness of peer verbal encouragement and investigate the role of other factors, such as social support and motivation, in the context of CrossFit fitness assessment.

While this research presents original data, it is important to acknowledge several limitations. The study did not distinguish between experienced and novice coaches, possibly restricting the applicability of the findings to the broader coach population. Caution is advised when extrapolating the study’s conclusions to include all coaches. Future research should deliberately investigate diverse coach backgrounds and expertise levels to foster a more comprehensive understanding of the multifaceted coach influence landscape. Another limitation of this study lies in the absence of control over the quantity or intensity of the VE expressions delivered by each peer or the coach. The study did not control for the volume of VE, and it is noteworthy that higher volumes of VE might be correlated with enhanced performance. Therefore, the potential impact of varying levels of VE intensity on participants’ responses remains unexplored, introducing a limitation to the study’s ability to draw nuanced conclusions regarding the relationship between VE and performance outcomes. Future research with explicit control over VE volume could provide deeper insights into this aspect.

## 5. Conclusions

In conclusion, this study demonstrated that VE can significantly improve 1-RM strength and functional endurance in the context of physical fitness CrossFit assessments. The study showed that VE had a stronger impact compared to NVE. Participants performed better with PVE than with other encouragement conditions. Furthermore, participants reported higher RPE and FS scores after receiving both PVE and CVE. These findings suggest that VE can be an effective tool for improving performance in CrossFit testing and training.

## Figures and Tables

**Table 1:** A detailed overview of the counterbalancing procedures employed in the study.

Week	Group	Session	Condition			Test Order					
			NVE	CVE	PVE	1-RM Squat	1-RM Deadlift	1-RM Bench Press	8MTT	RPE	FS
1	1	Mond.	×			1	2	3	4	5	6
	2	Tues.		×		3	1	2	4	5	6
	3	Wed.			×	2	3	1	4	5	6
2	1	Tues.		×		2	3	1	4	5	6
	2	Wed.			×	1	2	3	4	5	6
	3	Mond.	×			3	1	2	4	5	6
3	1	Wed.			×	3	1	2	4	5	6
	2	Mond.	×			2	3	1	4	5	6
	3	Tues.		×		1	2	3	4	5	6

Mond., Monday; Tues., Tuesday; Wed., Wednesday; NVE, No Verbal Encouragement; CVE, Coach Verbal Encouragement; PVE; Peer Verbal Encouragement; 8MTT, 8 min Time Trials; RPE, Rating of Perceived Exertion; FS, Feeling Scale.

## Data Availability

Data is available upon reasonable request from the first author.
